# A Social Marketing Intervention to Improve Treatment Adherence in Patients with Type 1 Diabetes

**DOI:** 10.3390/ijerph18073622

**Published:** 2021-03-31

**Authors:** Citlali Calderon, Lorena Carrete, Jorge Vera-Martínez, María Esther Gloria-Quintero, María del Socorro Romero-Figueroa

**Affiliations:** 1 Tecnologico de Monterrey, Business School, Toluca, Estado de Mexico 50110, Mexico; lcarrete@tec.mx; 2 Tecnologico de Monterrey, Business School, Tlalpan, Ciudad de Mexico 14380, Mexico; jorge.vera@tec.mx; 3 Endocrinología Pediátrica HGR 251, IMSS, Metepec, Estado de Mexico 52148, Mexico; megloriq@gmail.com; 4 Centro de Investigación en Ciencias de la Salud, FSC, Universidad Anáhuac Campus Norte, Naucalpan de Juárez 52786, Mexico; sromero61@hotmail.com

**Keywords:** social marketing, social representations theory, health belief model, shared beliefs, misconceptions, type 1 diabetes, T1DM, intervention, adolescents

## Abstract

This research explores if a social marketing intervention model based on social representations theory and the health belief model can generate changes regarding treatment adherence and improve patient self-efficacy. As a pilot, a test–retest field quasi-experiment was designed to evaluate the intervention model with type 1 diabetes (T1DM) patients of families with 8- to 17-year-old children. The intervention model was designed to clarify misconceptions, increase awareness of the benefits of following doctors’ treatments and improve patients’ self-efficacy. In-depth interviews were carried out to gain a richer understanding of the intervention’s effect. The pilot intervention generated a favourable change in shared misconceptions, individual health beliefs, glycaemic control and declared treatment adherence. This paper contributes to the social marketing literature and public health by providing early support for the theoretical assumptions regarding the role of shared misconceptions in physiological and behavioural outcomes for patients with T1DM. Contrary to previous studies, instead of only focusing on individual beliefs, this study incorporates shared beliefs between patients and caregivers, generating more comprehensive behavioural change.

## 1. Introduction

The International Diabetes Federation [1] estimates that over 1,110,100 children and adolescents globally live with type 1 diabetes (T1DM). People with diabetes have a high probability of developing serious heart ailments and infections. Following long-duration diabetes, microvascular and macrovascular complications occur [2]. Currently, T1DM cannot be prevented, although it can be controlled by adhering to a prescribed treatment protocol (insulin, diet, exercise and self-monitoring). Children with T1DM can live a long and healthy life if the condition is detected early and they adhere to complex, multicomponent regimens [3]. Unfortunately, children and adolescents do not follow all of their physicians’ directives either because of the complexity of regimens [4] or because, due to their ages, they are unable to understand the consequences of not following physicians’ instructions [5].

Several authors in the field of psychology have applied individual belief perspectives to the analysis of attitudes and behaviours of patients with diabetes [6,7]. However, analysis of the effects that shared beliefs (specifically misconceptions) have on treatment adherence, involving social marketing interventions, is still lacking [8]. The strong focus on individual health beliefs as determinants of complex chronic illnesses partially impacts several of the expected behaviours [9]. By including misconceptions that affect the individuals’ cognitive, behavioural and emotional states, this paper generates a better understanding of the determinants of behaviour change [10].

Drawing upon the relevant literature of social marketing [11,12], this study developed a pilot intervention aimed at clarifying shared misconceptions, increasing awareness of the benefits of following the treatment and improving youngsters’ self-efficacy. The concept of social marketing recognises that each person has the option to refuse a behaviour change; thus, a strategy that clearly states the benefits and value of voluntary behavioural change is necessary [13]. To develop a more effective intervention to enhance public health and social well-being, it is necessary to appropriately define marketing principles and techniques, such as environmental scanning, segmentation and the 4Ps [14].

Using a theory is also a critical component of the social marketing planning process [15,16]. Thus, this study uses Social Representations Theory (SRT) as its intervention framework. This theory helps explain the role of shared misconceptions in modifying or maintaining undesirable behaviours [17,18]. SRT is complemented with the Health Belief Model (HBM), a common conceptual framework applied to health issues in the social marketing literature [19,20]. To the best of the authors’ knowledge, no enquiry has integrated the two theories to demonstrate their applicability to promote behavioural change [17].

The current work took place in Mexico where the International Diabetes Federation [1] estimates that there are 26,578 children aged under 19 with T1DM. This segment of the population generates costs that represent 90.2% of the average Mexican annual household budget [21]. Therefore, improving the patients’ quality of life would help reduce pressure for families and health institutions. In the next section, the two theories used as a framework for the design of social marketing intervention are described.

### 1.1. Social Representations Theory

SRT stipulates that a social group produces a shared understanding of reality that creates a common context and guides interactions within the community. Sammut et al. define social representations as “systems of knowledge, or forms of common sense, that human subjects draw upon in order to make sense of the world around them and to act towards it in meaningful ways” [18]. These authors declare that “a social representations approach offers an empirical utility for addressing numerous social concerns such as social order, ecological sustainability, (…) health and social marketing”. SRT has been used to address social problems such as organ donation [22] and resistance to buy products with environmental and ethical claims [23]. However, the theory has rarely been used in social marketing issues. Based on it, Calderón [8] argues that when a group accepts misconceptions as “the truth”, they may generate behaviours that affect social welfare. Regarding TIDM, shared misconceptions about it and its treatment may inhibit adherence to doctors’ instructions. This is why SRT might be suited to solving this problem and will be studied alongside individual beliefs.

### 1.2. Health Belief Model

The HBM is a health behaviour change model where the likelihood of someone exhibiting a health behaviour is determined by five individual perceptual dimensions about beliefs: severity, benefits, barriers, susceptibility and self-efficacy [24]. In social marketing, the HBM has been used to design health campaigns or interventions aimed to promote human papillomavirus vaccination among young women [19], adolescent reproductive health [25], smoking cessation in prisons [26] and predicting the likelihood of healthy eating among college students [27]. Regarding diabetes, scholars have examined the role of health beliefs in adherence to treatment, with the five dimensions proving to be significant [28,29]. In other studies, the HBM has not been conclusive in explaining the lack of behavioural change [30,31].

Since the HBM is not enough in all cases to explain behavioural changes, this study integrates it and SRT to modify individual and social beliefs to favour treatment adherence and improve patient self-efficacy. In the following section, the social marketing pilot intervention is described and research hypotheses are formulated.

### 1.3. The Social Marketing Pilot Intervention Model and Hypotheses

The six criteria suggested by Stead et al. [12] for a social marketing programme were considered when planning the pilot intervention for the target audience.

#### 1.3.1. Behaviour Change Goal

The pilot intervention is aimed to clarify misconceptions shared by patients with T1DM and their primary caregivers to achieve better adherence to medical treatment (insulin, diet, exercise and self-monitoring). Informing patients and caregivers about the advantages of treatment adherence and improving self-efficacy, could result in a better glycaemic control (GC).

#### 1.3.2. Audience Research

To identify the shared misconceptions about T1DM in Mexico to be addressed in the intervention, the results obtained by Calderon in 2019 [8] were analysed and, with the assistance of a paediatric endocrinologist, were clustered into five categories described in Table 1: (1) general fallacies about T1DM, (2) insulin, (3) diet, (4) exercise and (5) self-monitoring.

#### 1.3.3. Segmentation

The target audience was minors diagnosed with T1DM and their primary familial caregivers. Participants were recruited from the Paediatric Endocrinology office of the Mexican Social Security Institute (IMSS) in Metepec, Mexico. The IMSS is the largest social security institution in Latin America. Details of the sample are given in the methodological section.

#### 1.3.4. Exchanges

To motivate behaviour change, subjects were informed of what they would receive in exchange for adherence to treatment. For children, this would mean living normally and eating whatever they want without health consequences. For primary caregivers, it may generate greater peace of mind knowing that their children are self-reliant. In this sense, their efforts will be rewarded at the personal and family levels.

#### 1.3.5. Competition

The main competitive force that participants face to treatment adherence is comfort as the regime involves following a complex, scheduled scheme (e.g., measuring glucose, insulin injection, exercise and carbohydrate counting). Another competitive force is Mexican dietary culture; the traditional intake includes a large number of carbohydrates, and all social gatherings are centred around food.

#### 1.3.6. Four Ps of the Social Marketing Mix

*Product:* The desired behavioural change and associated benefits were defined. Here, the health-related behaviour change of following the prescribed medical treatment represents the product definition. An endocrinologist who is highly experienced in T1DM and one of the current authors defined the benefits valued by the targeted audience. The children valued: (a) being able to live normally, (b) eating what they enjoyed and (c) feeling good physically, mentally and emotionally. The primary caregivers valued: (a) decreasing their fear, (b) reducing the occurrence of future sequelae, (c) seeing their children fare better and (d) knowing how to act in various circumstances.

*Pricing:* For this research, the costs (e.g., financial, emotional, psychological or time outlays) may be seen as external or internal factors that affect the audience (e.g., the lack of proper health care facilities, the belief that fate causes illness and the lack of skills to follow a treatment regime).

*Place:* This concept refers to where and when the target audience would learn the desired behaviour. From diverse options, the participants selected the most convenient facility to increase access and make locations more appealing [32].

*Promotion:* This includes communication strategies. An eight-session free workshop titled *Type 1 Diabetes and I* was prepared. Each session had a duration of two hours over four months (August 2017 to November 2017). Print material from the sessions delivered specific educational messages/information regarding T1DM (generalities, insulin, diet, carbohydrate counting, physical exercise and the ways in which to respond to those who spread misconceptions). Real, deliverable and near-term benefits were emphasised during the sessions. Creative elements were also included in the workshop in the form of Pink Panther; humour is a strategy used to target youngsters [32]. A nutritionist and a paediatric endocrinologist delivered presentations to strengthen the message.

Hence, the social marketing intervention model (SMI-T1DM) described above was conceptualised as the explanatory factor that should affect expected behavioural outcomes. Thus, the proposed dependent variables were elements embedded in those expected behavioural outcomes of such an intervention model. Therefore, the dependent variables implemented for this study were the following: (Y1) the correctness of the shared misconceptions (SM); (Y2) the correctness of the individual health beliefs (HBM); (Y3) the appropriateness of the personal glycaemic control (GC); and (Y4) the declared treatment adherence (DTA) level. Consequently, if such an intervention model, based on the elements outlined above, has the expected effectiveness, support for the following hypotheses could be obtained from this pilot test:

**Hypotheses** **1.** *The social marketing intervention model (SMI-T1DM) will generate a favourable change in shared misconceptions.*

**Hypotheses** **2.** *The social marketing intervention model (SMI-T1DM) will generate a favourable change in individuals’ health beliefs.*

**Hypotheses** **3 (H3a).** *The social marketing intervention model (SMI-T1DM) will generate a favourable change in glycaemic control.*

**Hypotheses** **3 (H3b).** *The change in individual health beliefs (HBM) is associated with the change in glycaemic control.*

**Hypotheses** **4.** *The social marketing intervention model (SMI-T1DM) will generate a favourable change in declared treatment adherence.*

## 2. Methodology

**Figure 1:**
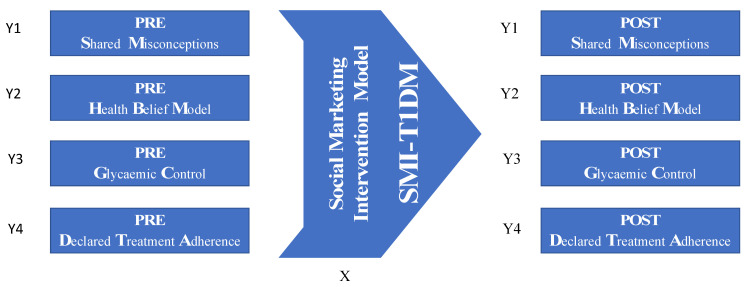
Scheme representing the research procedure.

A test–retest field quasi-experiment was designed to evaluate the pilot intervention organised for the treatment adherence of T1DM patients. This was accompanied by a qualitative process evaluation to gain a richer understanding of the intervention’s effect. Figure 1 represents the research procedure.

With prior authorisation from the IMSS local Committee for Ethics and Health Research, 30 families with children between 8 and 17 years old seeing a doctor from the organisation’s Paediatric Endocrinology office (Metepec, Mexico) were invited. The research group was selected through nonprobabilistic sampling of consecutive cases. The inclusion criteria were children who are between 8 and 17 years old with T1DM, insulin-dependent, who can read and write (both patient and primary caregiver), either gender, and who agree to participate through parents signing an informed consent form and children, who have a glucometer, signing a letter of agreement.

The final research group comprised 12 families (24 individuals among patients and primary caregivers) who met the inclusion criteria and consistently attended the four-month-long educational workshop. It is important to state that patients with T1DM are a hard-to-reach population because they make up 5–10% of everyone with diabetes, while T2DM accounts for around 90% of all cases of diabetes [33]. Despite the small sample size, Monte Carlo simulations have shown that when comparing very small samples of n = 5, n = 7 and n = 9, the Wilcoxon signed-rank test (and other nonparametric tests) is powerful enough to produce reliable results [34].

The shared misconceptions that were identified in the preliminary analysis were used to design a self-administrated questionnaire to assess whether the children and primary caregivers harboured these misconceptions. The design of the patient questionnaire incorporated four sections of open questions concerning (1) personal and treatment data, (2) self-image, (3) beliefs and knowledge about T1DM and its treatment and (4) support from and influence of external sources. Here are some examples of questions asked to identify misconceptions: “Why do you think you got T1DM?”, “Do you think T1DM can be cured?”, “What treatment do you think a person with T1DM should follow?”, “What are the effects of insulin?”, “Which diet do you think should an individual with T1DM follow?”, and “Can a person exercise when having T1DM?”. The questionnaire directed at primary caregivers also included four segments of open questions about (1) personal and treatment data, (2) description of their children, (3) beliefs about and knowledge of T1DM and its treatment and (4) support from and influence of external sources. The misconceptions were codified as 1 = absence and 0 = presence. During the first workshop session, patients handed over their Haemoglobin A1c laboratory results and blood glucose monitor, allowing researchers to download the measurements of the last fourteen days and obtain the variables of GC. The individual health beliefs of patients and their declared treatment adherence (DTA) were also measured at the first session.

The data obtained from the questionnaires became the basis for the content and design of the educational workshop to eliminate misconceptions. The questionnaire was reapplied to the same individuals at the end of the workshop (session 8) to determine whether misconceptions were eliminated. The glycaemic variables were measured again, and in-depth post-session interviews were conducted to gain a richer understanding regarding their perceptual changes about treatment adherence. The test–retest quasi-experiment was employed to compare any deviation in the dependent variables and analyse whether a potential change could be attributed to the social marketing pilot intervention.

The six intermediate sessions of the workshop focused on the following topics: general beliefs about T1DM (session 2), consequences of T1DM and the effects of insulin (session 3), misconceptions about diet (sessions 4 and 5), physical exercise (session 6) and how to respond to people when they spread false information about T1DM (session 7). The misconceptions related to self-monitoring were addressed in all the sessions because they are connected to the other themes, including insulin, diet and exercise.

### 2.1. Measurements

#### 2.1.1. Individual Health Beliefs (HBM)

A slightly modified version of the scale HBM-T1DM developed in 2018 by Calderón et al. [29], used to measure individual health beliefs, was administrated to the patients during the test and retest sessions to measure the dimensions of the HBM. The modified instrument comprised 20 items associated with four of the five dimensions of the HBM using a 6-point Likert scale ranging from “strongly disagree” to “strongly agree”. The dimension of perceived susceptibility was not evaluated in this research because increasing youngsters’ perception of vulnerability to possible sequelae was considered to be a delicate issue for the children.

Given that our research participants were minors, nine of the items were rewritten in a simple language with the assistance of a paediatric endocrinologist. Two children with T1DM were asked to read all of the survey and indicate whether they understood what was being asked in each question. Table 2 presents the phrasing of each item and the dimension evaluated.

#### 2.1.2. Glycaemic Control

Five clinically observable metrics on GC were measured during the test and retest sessions.

1. Blood glucose monitoring frequency (BGMF): a T1DM patient should make at least three daily assessments—more measurements imply better control. The average of daily measurements of patients during the previous 14 days is the daily blood glucose monitoring frequency.

2. Blood glucose average (BGA): data from the previous 14 days on the concentration of glucose in the blood were averaged.

3. Hypoglycaemia frequency (HypoF): the number of times that the glucose measurement was lower than 70 mg/dL during the previous 14 days was computed.

4. Hyperglycaemia frequency (HyperF): the number of times that the glucose measurement was higher than 249 mg/dL during the previous 14 days was calculated.

5. Haemoglobin A1c levels (HbA1c): the HbA1c test reflects the average levels of blood sugar over the past two and three months. The higher the HbA1c level, the poorer the blood sugar control and the higher the risk of health complications from diabetes.

#### 2.1.3. Declared Treatment Adherence

To measure the perceived treatment adherence, the following five items were designed on a 5-point scale in which 5 represented “always” and 1 signified “never”: “I follow my doctor’s instructions exactly when I inject my insulin”; “I follow the diet prescribed by my doctor and/or nutritionist”; “My exercise regime reflects my doctor’s instructions”; “I measure my glucose exactly as my doctor has instructed” and “I follow all my doctor’s instructions precisely”. These variables indicate the patients’ perception of the extent to which they were adhering to their treatment protocol during the study.

## 3. Results

The final sample comprised 12 families (24 individuals; 12 patients and 12 caregivers: nine mothers, two grandmothers and one grandfather). All the children were patients of the same doctor from the IMSS Paediatric Endocrinology office. The children’s average age was 13 (range = 11–15, SD = 1.5)—four were male and eight were female. The average number of years since diagnosis was three (range = 1–8, SD = 2).

### 3.1. Changes in Shared Misconceptions

The McNemar test was employed to analyse whether there was a significant modification of the shared misconception (SF) before and after the social marketing intervention. This nonparametric statistical test used for binary nominal data assumes two per two contingency tables with a dichotomous trait. It uses matched pairs of subjects to determine whether the row and column marginal frequencies are equal/unequal [35]. Results suggest a statistically significant decrease (in both patients and caregivers) in misconceptions across almost all categories: T1DM, insulin, diet and self-monitoring (Table 3). Exercise was the sole classification that did not register a significant modification because only 6 of the 24 participants had initial misconceptions regarding exercise; however, the misconceptions were clarified during the workshops. Thus, Hypothesis 1 can be tentatively accepted.

### 3.2. Variations in Individual Health Beliefs

The patients’ answers of the 20 items of the HBM-T1DM scale were clustered into their respective HBM dimensions (self-efficacy, barriers, benefits and severity). Each dimension was averaged and analysed using the Wilcoxon test to identify whether there was a significant change between the responses given before and after the intervention sessions. This nonparametric test assesses the magnitude of the difference between paired observations and can be used with small samples [36]. All four dimensions reported statistically significant changes (Table 4). The tally of all dimensions, barring barriers, increased as expected. Therefore, there is information that may support Hypothesis 2.

### 3.3. Modifications in Glycaemic Control

The Wilcoxon test was performed again to verify the differences in the medically observable variables of GC. All the variables presented significant changes in the expected direction (Table 4). The frequency of monitoring blood glucose increased, and all the other variables decreased, indicating a tentative improvement in the patients’ health. Thus, Hypothesis 3a could be accepted.

To test Hypothesis 3b, Kendall’s tau-b correlation was used to measure whether there was an association between the differences found in the GC variables (test–retest) and the modification of the scores pertaining to the individual dimensions of HBM. The Kendall tau-b correlation coefficient is a nonparametric measure of association that does not assume a normal distribution and therefore can be used with small samples and ordinal variables [36]. In this analysis, the results exposed a significant correlation between the changes in the HBM dimensions and two GC variables: Hyperglycaemia Frequency (HyperF) and Haemoglobin A1c (HbA1c) (Table 5). This result is important because the HbA1c test represents the actual treatment adherence over the previous three months and is the best indicator of long-term diabetes control [37]. A negative correlation was registered between the HbA1c levels and the HBM dimensions of self-efficacy, benefits and severity; as the HbA1c levels decreased, these HBM perceptions increased. In the case of barriers, the correlation was positive, since when the HbA1c levels decreased, the perceived barriers also decreased. Therefore, these data suggest possible acceptance of Hypothesis 3b.

### 3.4. Differences in Declared Treatment Adherence

When the Wilcoxon test was used for DTA, none of the items resulted in statistically significant changes (Table 4). Instead of an increment in the perception of attachment to the treatment, the results presented a decrease. Therefore, the adolescents were questioned using in-depth interviews to understand this decline. Out of the twelve patients, nine declared that they believed they were following all the necessary recommendations before the workshop. However, after the educational workshops, their perceptions adjusted. Even if Hypothesis 4 was rejected, the finding was interesting since the elimination of the misconceptions engendered greater awareness about the lack of treatment adherence.

### 3.5. Qualitative Support

This section presents a summary of verbatim quotes obtained from the open questions of the questionnaire and the in-depth interviews. The data aid in understanding the changes observed in misconceptions, GC and treatment adherence. Verbatims concerning the beliefs related to the diabetes treatment beliefs that the patients and primary caregivers had before and following the intervention are presented (Table 6).

Related to glycaemic control, the following quotations are examples that allow appreciation regarding how the intervention made participants more aware of the importance of glucose monitoring:

“I am learning. I did not know how to take care of myself. I had been checking my glucose levels only once a day (…) Now that I check my glucose levels more, I’m better; I have less hypoglycaemia and less hyperglycaemia and my glycosylated haemoglobin level is lower too” (male, 15 years, 4 years 2 months with diabetes).

*“*Monitoring helps control all other parts of the treatment: insulin, diet and exercise” (grandmother of a girl, 3 years 9 months with diabetes).

Finally, the following verbatims delve into how children changed their perception of treatment adherence by learning more about T1DM treatment:

“I could not understand that everything must work in coordination. It is not about eating and exercising a lot; it is more about eating balanced and exercising properly, always checking glucose and injecting myself with the insulin my body needs” (male, 15 years, 1 year 3 months with diabetes).

“I do things now that I did not do before. I was not checking my glucose enough; I was not exercising at all, and I thought that my treatment was only insulin. I believed that exercise was harmful to me” (female, 15 years old, 3 years 4 months with diabetes).

## 4. Discussion

According to Rundle-Thiele et al. [16], alternative theories should be used in social marketing to accomplish higher explanatory and predictive capability. In line with this proposal, approaching health problems through the lens of the social representations theory (SRT) seems to be relevant when dealing with shared misconceptions that are hindering the change in behaviours pursued by social marketing. This pilot intervention provided some empirical evidence regarding the role of shared misconceptions on the psychological and behavioural responses of patients with T1DM. Several systematic reviews have tried to determine the effectiveness of different educational processes to improve metabolic control, hospitalisations, complications and quality of life in children with T1DM [38]. In most studies examining the effect of educational interventions on HbA1c, there was no evidence of greater effectiveness of educational interventions provided as part of standard care. Related to successful interventions, these were heterogeneous and included cognitive behavioural therapy, family therapy, skills training and general diabetes education. Most of the studies reported reduced use of health services, although less than half were statistically significant [39]. The effect of educational interventions on diabetes knowledge is unclear in half of the studies that reported significant improvements. Interventions that had variable effects on knowledge scores included diabetes camps, general diabetes education and cognitive behavioural therapy. In the area of self-care/adherence to the regimen, some studies reported a significant improvement. Successful interventions included general diabetes education and cognitive behavioural therapy [40]. More recently, there have been studies developed with young patients living with T1DM that have shown its effectiveness in achieving improvements in GC [41]. These studies have also had a personalised educational approach, akin to the present research, and integrated children and their caregivers in the intervention group. Consequently, we do not think that our intervention model is superior; however, it is a new approach that builds from theories on social marketing, SRT and the HBM.

The analysis showed a modification in all HBM dimensions; the perceptions of severity, benefits and self-efficacy increased, while perceived barriers decreased. The changes in individual beliefs improved the GC of the patients. This finding agrees with those of Brownlee-Duffeck et al. [28] and Calderón [29], where the dimensions of health beliefs were also significant in explaining the adherence to treatment of diabetes patients. In this intervention, the Blood Glucose Average (BGA), HyperF and HypoF diminished significantly and the frequency of blood glucose monitoring increased. Thus, better self-monitoring enhanced general GC, which resulted in fewer glucose fluctuations. As has been asserted by other scholars, the “self-monitoring of blood glucose concentration is associated with improved GC in patients with type 1 diabetes” [42]. Similarly, a statistically significant decrease was noted in the HbA1c variable. This finding complements the previous literature highlighting HbA1c as a good indicator of GC [43]. Furthermore, a considerable correlation was found between the changes in the dimensions of the HBM and decrease in the HbA1c values. These preliminary findings suggest that clarifying misconceptions shared by families and adjusting children’s individual beliefs towards T1DM (severity, benefits, barriers and self-efficacy) tend to decrease HbA1c.

When evaluating the association between the changes in the dimensions of the HBM and HbA1c laboratory results, it was found that perceived barriers and self-efficacy had the highest correlation (0.984 and −0.969, respectively). Apparently, greater adherence to treatment was achieved with lower perceived barriers. This outcome was consistent with Rosenstock et al.’s work [44], who described perceived barriers as a powerful dimension relative to other HBM dimensions. However, in contrast, Aldohaian et al. [31] indicate that perceived barriers had low incidence regarding cervical cancer screening tests. This discrepancy may reinforce the idea of combining SRT and the HBM to modify shared and individual beliefs to achieve desired behavioural changes.

The modification in the DTA did not yield a significant result. Most of the subjects stated that before the intervention they thought they were doing everything that was needed to lead a healthy life. However, after the educational workshops, apparently, their perceptions were transformed. This insight was identified through the postqualitative interviews. The readjustment of self-perceptions after an intervention has been documented in the literature [45].

We suggest a cautious interpretation of these preliminary findings given the exploratory nature of this social marketing intervention. First, probability sampling was not possible because of limited access to families that included 8- to 17-year-olds with T1DM (it is a hard-to-reach population). Additionally, participating families had to consistently attend the four-month-long workshop. As all the participants were preadolescents and adolescents, the stability of their glucose levels may have been affected by hormonal changes. It is plausible that their treatment adherence was also influenced by attitudinal elements related to their ages; adolescent patients tend to abandon their treatment [46,47]. Therefore, health promoters should identify innovative ways to engage young patients.

## 5. Conclusions

To the best of the authors’ knowledge, this is the first study to consider individual beliefs alongside shared misconceptions to influence behavioural outcomes of adolescent and preadolescent T1DM patients. The removal of individual and shared misconceptions alongside the improvement of capabilities/self-efficacy allowed participants to manage their complex regimen. This paper tentatively proposes a social marketing intervention model based on the HBM and SRT. Future experimental social marketing intervention studies are needed to determine the applicability of the model in adults with T1DM and health problems where misconceptions are common. Previous research has suggested that patients with glaucoma [48], heart failure [49], type 2 diabetes [50] and HIV [51] do not adhere to treatment due to misconceptions. To reduce chronic disease mortality by improving adherence rates, identifying and addressing misconceptions that patients hold about treatments is necessary [52]. In future studies, it would be important to test (individually) each of the elements forming part of this intervention model. This would make it possible to determine which of such elements may have the greatest effect on clarifying misconceptions towards medical treatments. Improving adherence rate in chronic diseases could increase the patient’s quality of life and decrease economic pressure, both for families and health institutions.

## Figures and Tables

**Table 1:** Frequent Type 1 Diabetes (T1DM) misconceptions in Mexico.

Type	Misconception	Description
General T1DM	T1DM comprises immediate consequences and is a death sentence	T1DM encompasses immediate consequences such as amputations, blindness and kidney failure and eventually leads to death.
	T1DM can be prevented	T1DM is the consequence of an unhealthy lifestyle. It is an illness that afflicts old, overweight and sedentary people. It can also be caused by fright, stress, or consuming high amounts of sugar.
	T1DM can be cured	Healthy food, traditional remedies or alternative therapies can cure T1DM.
	T1DM is hereditary	T1DM is inherited from people with type 2 diabetes.
Insulin	Insulin has harmful effects	Insulin shots can cause blindness, amputations or death through heart or kidney disease.
	People without diabetes do not need insulin in their bodies	Insulin is a medication that was created for diabetes.
	T1DM can be treated only with insulin	Insulin is enough to control diabetes. It is the only important element in the treatment.
	Insulin is not necessary	Nothing will happen if a person with T1DM does not get insulin injections.
Diet	People with T1DM need to follow a special diet	People with T1DM cannot eat the same food as people who do not have the condition. They should never eat sweets, but fruits can be freely eaten. People with T1DM can eat as much “light food” as they want.
	T1DM can be treated with a diet only	Alimentary lifestyle changes are enough to control T1DM. Medication or other treatment is not necessary.
Exercise	T1DM can be treated with exercise only	Physical lifestyle changes are enough to control diabetes.
	Individuals with T1DM cannot exercise or participate in sports	Exercising is very dangerous for people with diabetes and should never be undertaken.
Self-Monitoring	Self-monitoring is not necessary	If a person with T1DM injects their insulin and follows a diet, it is not necessary to check the glucose level.
	Frequent self-monitoring is not necessary	If a person with T1DM does not feel ill, it is not necessary to check the glucose levels in the blood.

Adapted with permission from Calderon (2019) [8].

**Table 2:** Adapted Health Belief Model (HBM)-T1DM items and dimensions.

Dimension	Item Number	Item
self-efficacy	1	I can follow my diet even when I eat with other people who do not have diabetes.
self-efficacy	5	If my blood sugar drops, I can go back to an adequate level.
self-efficacy	10	If my blood sugar goes up, I can go back to an adequate level.
self-efficacy	14	I can calculate the correct amount of insulin that I should inject into myself according to the food I have eaten.
self-efficacy	17	I can measure my blood sugar 3–6 times a day.
barriers	2	It is hard to follow my diet when I eat outside my house.
barriers	6	Controlling my diabetes affects my activities at school.
barriers	9	Controlling my diabetes affects how I can enjoy my free time.
barriers	13	Having diabetes complicates my life.
barriers	18	Controlling my diabetes affects how I can hang out with friends.
benefits	3	Following a diet helps me control my diabetes.
benefits	7	Measuring my blood sugar regularly helps me control my diabetes.
benefits	11	Exercising regularly helps me control my diabetes.
benefits	16	Injecting the right amount of insulin helps me control my diabetes.
benefits	19	I can avoid future complications if I follow the doctor’s instructions.
severity	4	Going into a coma because of low blood sugar is serious.
severity	8	Having blurred vision is serious.
severity	12	Going into a coma for high blood sugar is serious.
severity	15	Developing kidney damage is a serious problem.
severity	20	Developing circulation problems in the feet is a serious problem.

**Table 3:** McNemar’s test for differences between binary variables test/retest contrasts.

		T1DM	Insulin	Diet	Exercise	Self-Monitoring
Test/retest Patients * misconceptions	Sig. (2-tailed)	0.000	0.000	0.002	0.250	0.002
Test/retest Caregivers ** misconceptions	Sig. (2-tailed)	0.002	0.001	0.004	0.250	0.002

Notes: *p*-value under 0.025 implies significant differences between both measurements. Binomial distribution. The period between measurements pre–post was four months. * n = 12 patients, ** n = 12 caregivers.

**Table 4:** Wilcoxon signed-rank tests for test/retest contrasts.

Test/retest HBM dimensions average			Self-efficacy	Barriers	Benefits	Severity
	Z		−3.063 ^b^	−3.062 ^c^	−3.063 ^b^	−3.062 ^b^
	Sig. (2-tailed)		0.002	0.002	0.002	0.002
Test/retest Glycaemic control		BGMF ^1^	BGA ^2^	HyperF ^3^	HypoF ^4^	HbA1c ^5^
	Z	−3.065 ^b^	−3.059 ^c^	−2.969 ^c^	−2.890 ^c^	−3.062 ^c^
	Sig. (2-tailed)	0.002	0.002	0.003	0.004	0.002
Test/retest Declared treatment adherence		Insulin	Diet	Exercise	Self-monitoring	General
	Z	−1.265 ^b^	−1.897 ^b^	−1.645 ^b^	−1.588 ^b^	−1.613 ^b^
	Sig. (2-tailed)	0.206	0.058	0.100	0.112	0.107

Notes: *p*-value under 0.025 implies significant differences between both measurements, ^1^ Blood Glucose Monitoring Frequency (BGMF), ^2^ Blood Glucose Average (BGA), ^3^ Hypoglycaemia frequency (HypoF), ^4^ Hyperglycaemia frequency (HyperF), ^5^ Haemoglobin A1c levels (HbA1c). ^b^ Based on negative ranks. ^c^ Based on positive ranks. The period between measurements pre–post was four months. n = 12 patients.

**Table 5:** Kendall’s Tau-b correlation coefficient for Health Belief Model (HBM) dimensions and glycaemic control.

			Test/Retest HBM Dimensions Average Difference			
			Self-Efficacy	Barriers	Benefits	Severity
Test/retest glycaemic control difference	BGMF	Correlation Coefficient	−0.254	0.254	−0.24	−0.175
		Sig. (2-tailed)	0.265	0.265	0.295	0.444
	BGA	Correlation Coefficient	−0.4	0.431	−0.419	−0.431
		Sig. (2-tailed)	0.073	0.054	0.062	0.054
	HyperF	Correlation Coefficient	−0.618 **	0.635 **	−0.571 *	–0.738 **
		Sig. (2-tailed)	0.009	0.007	0.017	0.002
	HypoF	Correlation Coefficient	0.272	−0.272	0.223	0.153
		Sig. (2-tailed)	0.252	0.252	0.351	0.519
	HbA1c	Correlation Coefficient	−0.969 **	0.984 **	−0.898 **	−0.813 **
		Sig. (2-tailed)	0.000	0.000	0.000	0.000

Notes: ** Correlation is significant at the 0.01 level (2-tailed). * Correlation is significant at the 0.05 level (2-tailed). n = 12 patients.

**Table 6:** Beliefs before and after the intervention.

	Response before Intervention	Response after Intervention
What is type 1 diabetes?	It’s a disease that occurs when you eat a lot of sugar and the blood fills with sugar (female, 11 years old, 2 months with diabetes). Congenital condition that derives from the transmission of hereditary genes from parents or grandparents (father of a girl, 4 years with diabetes).	It’s a disease in which the pancreas no longer produces insulin because the antibodies attack it. Hence, as there is no insulin, and insulin is the key with which glucose enters the cells, your cells starve (female, 11 years old, 3 years 6 months with diabetes).
Why do you think you got type 1 diabetes?	As I ate a lot of sweets in one week (female, 14 years old, 5 years with diabetes).	I did not get sick from inheritance or a scare, what happened is that my pancreas stopped producing insulin (male, 15 years, 1 year 3 months with diabetes).
Do you think you could have prevented getting type 1 diabetes?	Yes, eating a different diet and exercising (mother of a girl, 3 years with diabetes).	All participants answered “No”.
Do you think type 1 diabetes can be cured?’	Yes, taking good care of myself, following a diet, not eating sweets, eating more vegetables (male, 14 years old, 1 year 9 months with diabetes). Yes, eating healthy and exercising daily (mother of a boy, 1 year 3 months with diabetes).	All the participants answered ‘No’.
What is insulin?	Insulin helps me control my glucose levels, but it can blind me, still I need it … (female, 13 years, 1 month with diabetes). Insulin produces what the kidney or pancreas no longer produce (mother of a boy, 2 years with diabetes).	It’s a hormone produced by the pancreas so that the body can absorb glucose and be transported throughout the body (female, 14 years old, 2 years 9 months with diabetes).
Which diet do you think should be followed when an individual has type 1 diabetes?	Eat a lot of fruits and vegetables (female, 11 years old, 3 years 2 months with diabetes).	Have a balanced diet with carbohydrate counting and drink water (female, 13 years, 5 months with diabetes).
Is it helpful to monitor your blood sugar when you have type 1 diabetes?	No, it only helps me to know if I can go to sleep (female, 13 years old, 1 month with diabetes).	Yes, to know how much I have to inject and what I can eat (male, 15 years old, 4 years 2 months with diabetes).

## Data Availability

The data presented in this study are available on request from the corresponding author. The data are not publicly available due to privacy restrictions.
